# Skin regeneration is accelerated by a lower dose of multipotent mesenchymal stromal/stem cells—a paradigm change

**DOI:** 10.1186/s13287-020-02131-6

**Published:** 2021-01-25

**Authors:** Gertraud Eylert, Reinhard Dolp, Alexandra Parousis, Richard Cheng, Christopher Auger, Magdalena Holter, Ingrid Lang-Olip, Viola Reiner, Lars-Peter Kamolz, Marc G. Jeschke

**Affiliations:** 1 Sunnybrook Research Institute, Sunnybrook Health Sciences Centre, Toronto, Canada; 2 Division of Plastic, Aesthetic, Reconstructive Surgery, Medical University of Graz, Graz, Austria; 3 Institute of Medical Science, University of Toronto, Toronto, ON Canada; 4 Department of Psychiatry, Queen’s University, Kingston, Canada; 5 Institute of Biomaterials and Biomedical Engineering, University of Toronto, Toronto, Canada; 6 Institute of Biostatistics, Medical University of Graz, Graz, Austria; 7 Division of Cell Biology, Histology, Embryology, Gottfried Schatz Research Center, Medical University of Graz, Graz, Austria; 8 Coremed- Centre for Regenerative Medicine, Joanneum Research Forschungsgesellschaft mbH, Toronto, Canada; 9 Ross Tilley Burn Centre, Sunnybrook Health Sciences Centre, Toronto, Canada; 10 Division of Plastic and Reconstructive Surgery, Department of Surgery, Faculty of Medicine, University of Toronto, Toronto, Canada; 11 Department of Surgery, Division of Plastic Surgery, Department of Immunology, Director Ross Tilley Burn Centre, Sunnybrook Health Sciences Centre, Sunnybrook Research Institute, 2075 Bayview Ave., Graz, M4N 3M5 Austria

**Keywords:** Skin regeneration, Multipotent mesenchymal stromal/stem cells, Wound healing, Cell therapy, Tissue engineering, Integra, Umbilical cord mesenchymal stromal/stem cells, Experimental surgery, Skin substitutes

## Abstract

**Background:**

Multipotent mesenchymal stromal/stem cell (MSC) therapy is under investigation in promising (pre-)clinical trials for wound healing, which is crucial for survival; however, the optimal cell dosage remains unknown. The aim was to investigate the efficacy of different low-to-high MSC dosages incorporated in a biodegradable collagen-based dermal regeneration template (DRT) Integra®.

**Methods:**

We conducted a porcine study (*N* = 8 Yorkshire pigs) and seeded between 200 and 2,000,000 cells/cm^2^ of umbilical cord mesenchymal stromal/stem cells on the DRT and grafted it onto full-thickness burn excised wounds. On day 28, comparisons were made between the different low-to-high cell dose groups, the acellular control, a burn wound, and healthy skin.

**Result:**

We found that the low dose range between 200 and 40,000 cells/cm^2^ regenerates the full-thickness burn excised wounds most efficaciously, followed by the middle dose range of 200,000–400,000 cells/cm^2^ and a high dose of 2,000,000 cells/cm^2^. The low dose of 40,000 cells/cm^2^ accelerated reepithelialization, reduced scarring, regenerated epidermal thickness superiorly, enhanced neovascularization, reduced fibrosis, and reduced type 1 and type 2 macrophages compared to other cell dosages and the acellular control.

**Conclusion:**

This regenerative cell therapy study using MSCs shows efficacy toward a low dose, which changes the paradigm that more cells lead to better wound healing outcome.

**Supplementary Information:**

The online version contains supplementary material available at 10.1186/s13287-020-02131-6.

## Introduction

After a skin injury, skin regeneration and wound healing of the epidermis and dermis are crucial to lowering the risk of infections associated with high mortality [1]. Therefore, in wound treatment, skin substitutes play an important role and provide temporary or permanent wound coverage [2] if autologous, allo- or xenografting therapy is unavailable. Cellularized skin substitutes aim to mimic skin and are being developed having great potential [3–5] once they are commercially available. Many acellular skin substitutes are widely used [6]. Integra® is one of the most recognized scaffolds worldwide and is approved for acute as well as chronic wounds [7]. It is a synthetic biodegradable bilayer consisting of a bottom acellular dermal matrix—a porous crosslink of bovine type I collagen and shark cartilage—and an upper-protecting silicon layer. This acellular dermal regeneration template (DRT) provides a scaffold for endogenous cell ingrowth and dermal stroma synthesis following healing.

Clinical trials are being conducted to investigate wound healing using multipotent stromal cells as known as mesenchymal stem cells (MSCs) [1, 8] incorporated into Integra® (e.g., adipose, bone marrow, (burn-) skin-derived, umbilical cord). However, the critical cell dosing of MSCs is unknown and difficult to compare based on previous reports. Publications utilizing the DRT for wound healing include MSCs cell dosages that vary between 5000 and 2,000,000 cells/cm^2^ [9–11] and are tested on different models, such as rodents [11–15], pigs [9, 10, 16], and humans [17]. These studies were either given cell dosages once [9, 10, 16, 18] or multiple times [11] on acute [9, 10, 16, 18] and chronic wounds [17] either on partial [18] or full-thickness [9, 10, 16] (burn) wounds, making comparisons even more difficult.

However, we previously observed in an umbilical cord stem cell study using a bio-printer with direct cell depositioning onto burn wounds comparing to the cellularized DRT that even a lower dose regenerated the skin [10]. This raises the challenging question of which dose is optimal, as the general research hypothesis from these previous papers is that more cells lead to better wound healing outcomes.

The aim of this study was to determine the efficacy of low-to-high doses of MSCs incorporated into the DRT for wound healing and skin regeneration, applied once on full-thickness burn excised wounds.

## Material and methods

### MSC preparation

Umbilical cord mesenchymal stromal/stem cells (UC-MSCs) [19] were used based on the long history using perinatal tissue in (burn) wound care, its impressive healing capabilities [1, 20–22], and its easy accessibility. In addition, trials [22, 23] have shown their safety [22–24], their superior multipotent potential compared to other MSC sources [25], their excellent immunosuppressive properties with a low risk of graft-versus-host disease [26], and their potency under ischemic-like stress conditions [27].

MSCs were extracted from the stroma—Wharton’s Jelly from umbilical cords [28, 29], which we received from the Obstetrical and Gynecology Department at the Sunnybrook Hospital, cultured (Gibco™ DMEM, Thermo Fischer Scientific, enriched with 1% antibiotic-antimycotic solution, Gibco™, 1% L-Glutamine, Sigma Aldrich, and 10% fetal bovine serum, Gibco™ Life Technologies Corporation, USA), and expanded (until cell passage 3–4). Further, stem cell differentiation assays were performed to confirm the differentiation potential into the mesenchymal lineages (adipose, cartilage, and bone) [10], as recently described and shown using our published protocols in a parallel project using the same cells seeded on Integra® [10], both followed after confirming the paracrine in vitro effects of the extracted cells for wound healing in our lab as previously shown [29, 30].

Cells were sorted via flow cytometry (BD™ LSR II Flow Cytometer, BD Biosciences, Canada using FACSDIVA™, BD Biosciences, Canada using FlowJo™ software) for MSCs according to the International Society for Cellular Therapy [31] as previously described [10]. Live cells were selected and gated with the negative markers CD34−/CD11b−/CD45− (FITC) (Invitrogen), CD19−/HLA−DR− (AF700, PE-Cy7) (eBioscience), and positive markers were gated for CD73+ (PE) (eBioscience), CD90+ (BV510) (eBioscience), and CD105+ (APC) (eBioscience).

### Cell incorporation into the DRT Integra®

The commercially available DRT Integra® was used, because it has been demonstrated as a reliable cell carrier for tissue engineering [9, 12–16, 32], which allows cell ingrowth [33, 34] as well as cell differentiation [35].

As previously described [9], first, sorted UC-MSCs were resuspended and spun down. A cell count for viability was performed. Second, equal cell distributions for each wound treatment were transferred into 50 ml Falcon tubes containing + 25% of cells and 2 ml cell medium (Gibco™ DMEM, enriched with 1% antibiotic-antimycotic solution, 1% L-glutamine, and with 10% FBS). Third, the cells were resuspended and transferred into a petri-dish and homogenously pipetted with a multi-channel-pipette (VWR High Performance Signature™) on the acellular Integra® on top of the bovine collagen, with the silicone side facing down on a sterile cell culture disk. The cells were seeded on DRT, which builds connections with the wound bed after surgical placement. Each DRT was prepared with 200–2,000,000 cells/cm^2^ according to our experimental protocol. The acellular control was prepared similarly with a mix of PBS and DMEM. Importantly, the DRTs absorbed the entire volume of the cells and PBS suspensions. Both groups were then placed in the incubator at 37 °C at 5% CO2 until grafting on the pig. Shortly before surgery, the cellularized scaffolds were assessed under the microscope for floating cells indicating cell death and/or failure to integrate. No floating cells could be detected in either of the scaffolds, indicating full cell integration. From initial scaffold preparation until surgical grafting, less than 90 min of time had passed.

One Integra® scaffold with a cell density of 5000 cells/cm^2^ was assessed 12 h after cell incorporation and incubation at 37 °C at 5% CO_2_ using a confocal microscope. By imaging, cells were detected until a depth of 123 ± 21 μm, in the 1.3 mm thick scaffold, including the silicon bi-layer (Supplementary Figure 1A-D).

### Full-thickness burn porcine model

Yorkshire pigs [36] were used (*N* = 8) which possess similar anatomic and physiologic skin characteristics and comparable pigmentation to humans [16, 37, 38]. Large wound sizes did not allow spontaneous healing via contracture [39]. The model has been validated from other authors as a sufficient full-thickness burn excised wound model [16, 37, 38].

One week after being acclimatized and treated with preventive antibiotic for 5 days (ceftiofur injection daily), all eight 4-month-old male Yorkshire pigs, with a minimal weight 25 kg and length of 60 cm, were exposed to full-thickness burn injuries until the muscle fascia of multiple 5 × 5 cm wounds (TBSA of 25%) on the dorsal back after a standardized protocol under general anesthesia and analgesia (Buprenorphine 0.05 mg kg − 1 subcutaneous, ketamine 0.2 mg kg − 1 subcutaneous combined with atropine 0.5–1.0 mg depending on the heart rate, as well as isoflurane 5%/l/O_2_ intubation).

For wound infliction, a heated aluminum device (200 °C) was used for 20 s and digital force gauge (4.0 N, Mark-10 Corporation) (1 N = 1 kg m s − 2) (on day − 2). Further analgesia (tramadol 2–4 mg/kg/every 8 h orally) was administered regularly during the experiment. Full-thickness burn wounds were histologically confirmed [38] 48-h post-burn via punch-biopsy as described previously (on day 0) [10] using our published protocol [9, 10].

### Wound treatment

Full-thickness burn tissue excision and hemostasis were performed 48-h post-burn until the muscle fascia on the surgery day (day 0), and wounds were treated with the prepared cellularized DRT and the acellular control (Integra® alone). The scaffolds were additionally fixed via skin stapler on the wound edges. Regular wound dressing changes (2–3 times/week), as well as 4 mm tissue punch biopsies, were performed at determined timepoints. Wound dressing was applied using a layer of topical antibiotics (Polysporin®), fat-gauze (Jelonet®), multiple layers of gauze, as well as adhesive dressing (Tegaderm®), and a costume-made animal compression jacket (Fig. 2a).

### Presence of labeled cells on the wounds

Sorted UC-MSCs (1,000,000) were labeled with 6 μl of a lipid cell surface dye (DiO; Vybrant Cell Labeling Kit, eligible for flow cytometry, DiO yellow channel (V-22886) Abs 484(nm)/Em 501 (nm), FITC) [40]. Additionally, cell viability after labeling was performed according to the manufacturer’s protocol and assessed 12 h using Live/Dead® Viability/Cytotoxicity Kit, Invitrogen (Calcein 494/517 nm, Ethidium homodimer-1/DNA 528/617 nm). The labeled cells were incorporated with a density of 40,000 cells/cm^2^ into equally cut 5 × 5 cm meshed acellular DRT, and were grafted on full-thickness burn excised wounds on day 0. Full-thickness tissue biopsies were taken on days 2, 4, 7, and 9 at every dressing change from rotational quadrants of the wounds. The tissue biopsies were collagenased and analyzed via flow cytometry for detection of a double positive signal with DiO on CD90+ cells (BV510) (eBioscience). Labeled cells (CD90+, DiO) were present in the wound biopsy on the pigs until day 7 in a repeated experiment (Supplementary Figure 1E).

### Wound healing assessment

On day 28, photography and biopsies were taken from each wound center and fixed in formalin, followed by 70% EtOH. Paraffin-embedded slides were stained after protocols for Masson’s trichrome and immunohistochemistry. Antibodies used were CD11b (ab133357, rabbit monoclonal, Abcam), CD163 (ab87099, rabbit polyclonal, Abcam), CD31 (ab28364, rabbit polyclonal, Abcam), and aSMA (ab18415, monoclonal, Abcam), which were visualized via HRP polymer detection, followed by betazoid DAB chromogen kits (Biocare), before mounting and evaluation by light microscopy (LeicaDM 2000 LED). All histology samples were assessed on three different points on the epidermis and in the dermis, measuring in the same depth, from the epidermis 2000 μm into the dermis. Two blinded independent researchers evaluated each sample, and two blinded plastic surgeon clinicians evaluated the photography, being familiar with the chosen approach.

### Cell dose, statistical analysis

The arbitrary cell dose accounts for the MSC treatment per wound size (e.g., 200 cells/cm^2^ = 5000 cells/wound, 40,000 cells/cm^2^ = 1,000,000 cells/wound, and 2,000,000 cells/cm^2^ = 50,000,000 cells/wound).

The outcome of each wound healing parameter was analyzed descriptively (median, IQR) in the non-parametric data-set using Microsoft Excel. Graphical presentation was performed using GraphPad Prism Version 8.0. For the Supplementary Material for the dose-curves, the statistical program Python was used. Graphical illustrates is shown with a regression (of order 2) with the line-of-best-fit, and with a 95-confidence interval (Supplementary Material Figure 2).

### Ethical approval

This study was approved and performed in accordance with the guidelines and regulations of the Research Ethics Board (REB), Sunnybrook Health Science Centre (REB # 017-2011) (AUP # 16-600). It was executed accordingly in agreement with the Animal Policy and Welfare Committee of the University of Toronto, where veterinarian technicians monitored the procedure and wellbeing in routine safety and health checks. During the trials, no adverse events occurred. The investigated animals maintained their health during the entire experiment.

## Results

### Macroscopical wound healing

**Fig. 1:**
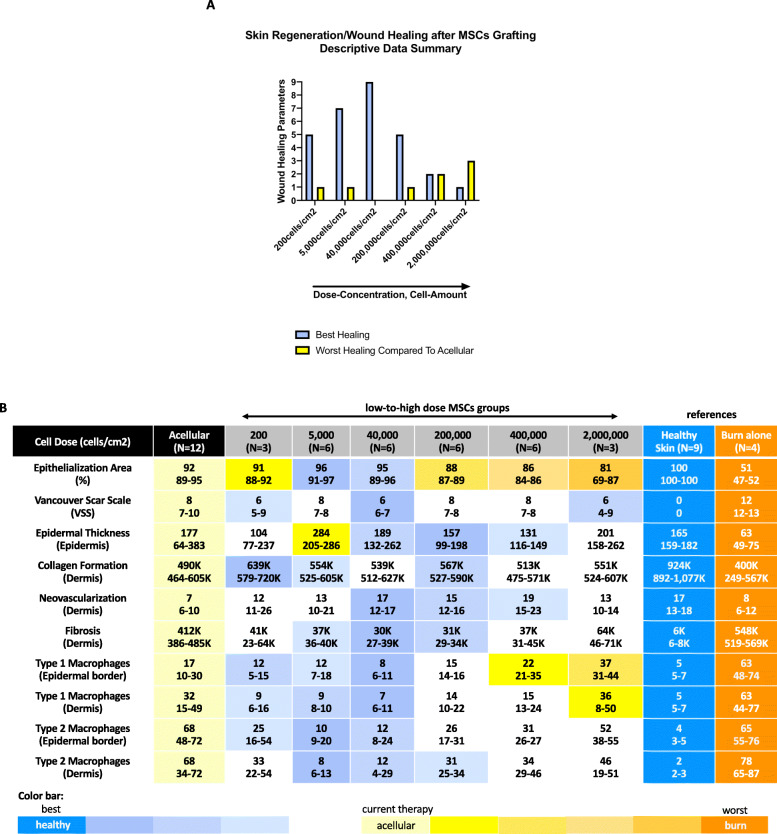
Descriptive data summary of the study, cell dose concentration. **a** Summary of the outcome measures of the dataset. **b** Data in median and interquartile range (IQR). K indicates 1000. Heat color map: dark blue indicates healthy skin as the physiologic and best condition. Lighter blue shades are first, second, and third best, respectively. Light yellow shaded color indicates the acellular control, which is the current treatment standard used in clinic. Everything from yellow to dark orange indicates a worst outcome compared to the acellular control. Orange indicates burn alone, the worst condition

**Fig. 2:**
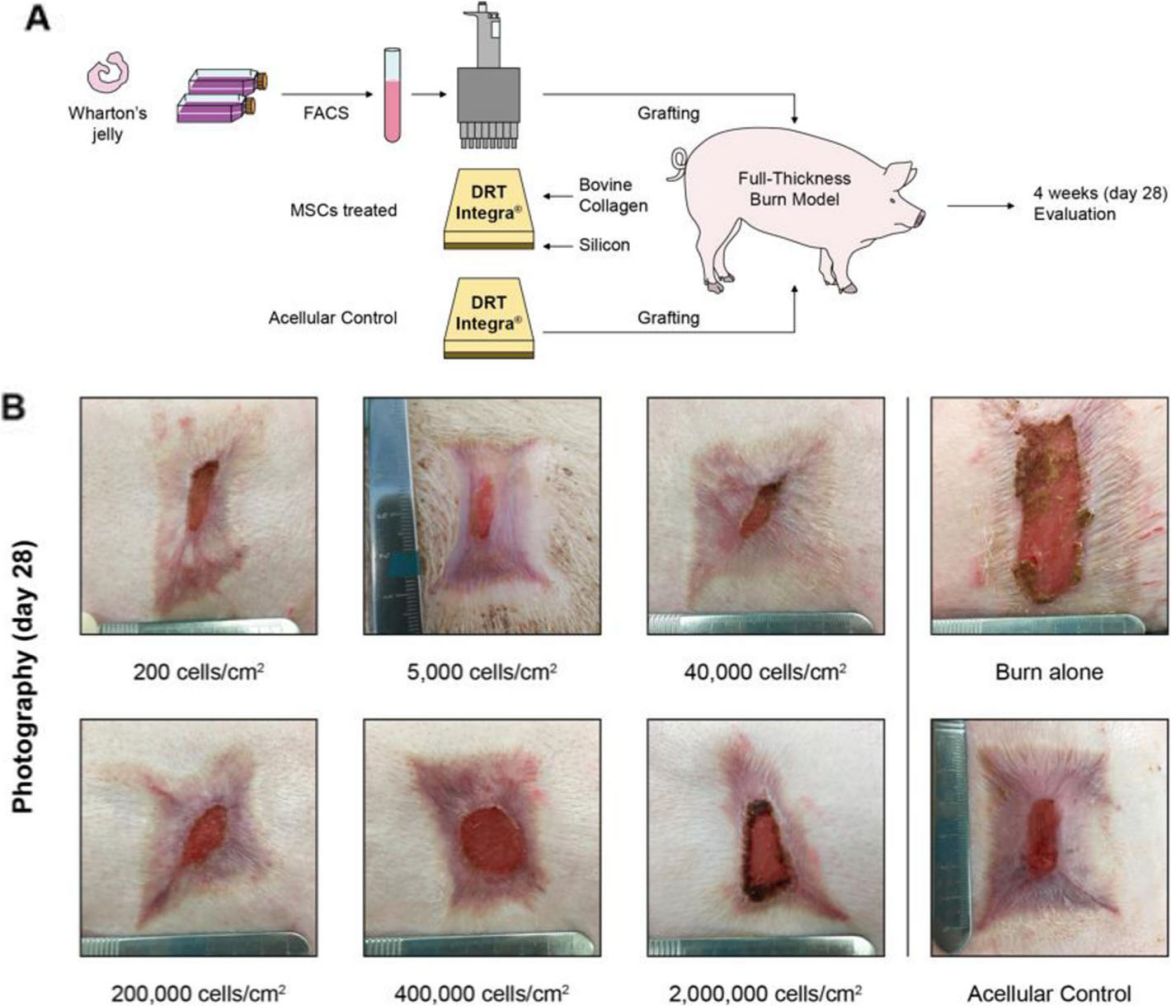
Overview experiment, macroscopical wound healing. **a** Overview of the experiments. **b** Photography of macroscopical wounds on day 28 from the initial 5 × 5 cm full-thickness burn excised wounds

Wound healing was assessed via photography after 4 weeks after treatment, as per the definition in the remodeling phase [41]. The epithelialization area per wound was calculated [(area without epithelialization in cm^2^ on day 28 × 100)/initial wound size in cm^2^ on day 0)]. The MSC-treated groups showed a median between 96 and 81% epithelialization compared to the acellular control with a median of 92% (IQR 89–95). The low dose group with 5000 cells/cm^2^ showed the fastest epithelialization with 96% epithelialization (IQR 91–97), followed by 40,000 cells/cm^2^ with 95% epithelialization (IQR 89–96). The lowest dose of 200 cells/cm^2^ and high doses of 200,000–2,000,000 cells/cm^2^ showed inferior wound healing compared to the acellular control with epithelialization between 81 and 91% (IQR 69–92) (Figs. 1c and 2b).

Scarring was assessed using the Vancouver Scar Scale (VSS, vascularity, pigmentation, pliability, and height), which is the most recognized and validated [42] scar scale [43, 44], and has been used previously for skin graft assessment [10, 44]. The MSC-treated group of 40,000 cells/cm^2^ showed the lowest scarring with a median VSS of 6 with the narrowest interquartile range (IQR 6–7). The highest dose of 2,000,000 cells/cm^2^ (IQR 4–9) and the lowest dose of 200 cells/cm^2^ (IQR 5–9) both had the same median VSS of 6. The other MSC-treated groups of 5000, 200,000, and 400,000 cells/cm^2^ showed a median VSS of 8 (all IQR 7–8), compared to the acellular control with the same median VSS of 8 (IQR 7–10). Overall the MSC-treated groups appeared less inflamed, with a more homogenous scar texture. The lowest and the highest dose had a sample size of *N* = 3, while the other dose groups had *N* = 6 (Figs. 1c and 2b).

### Epidermal regeneration

**Fig. 3:**
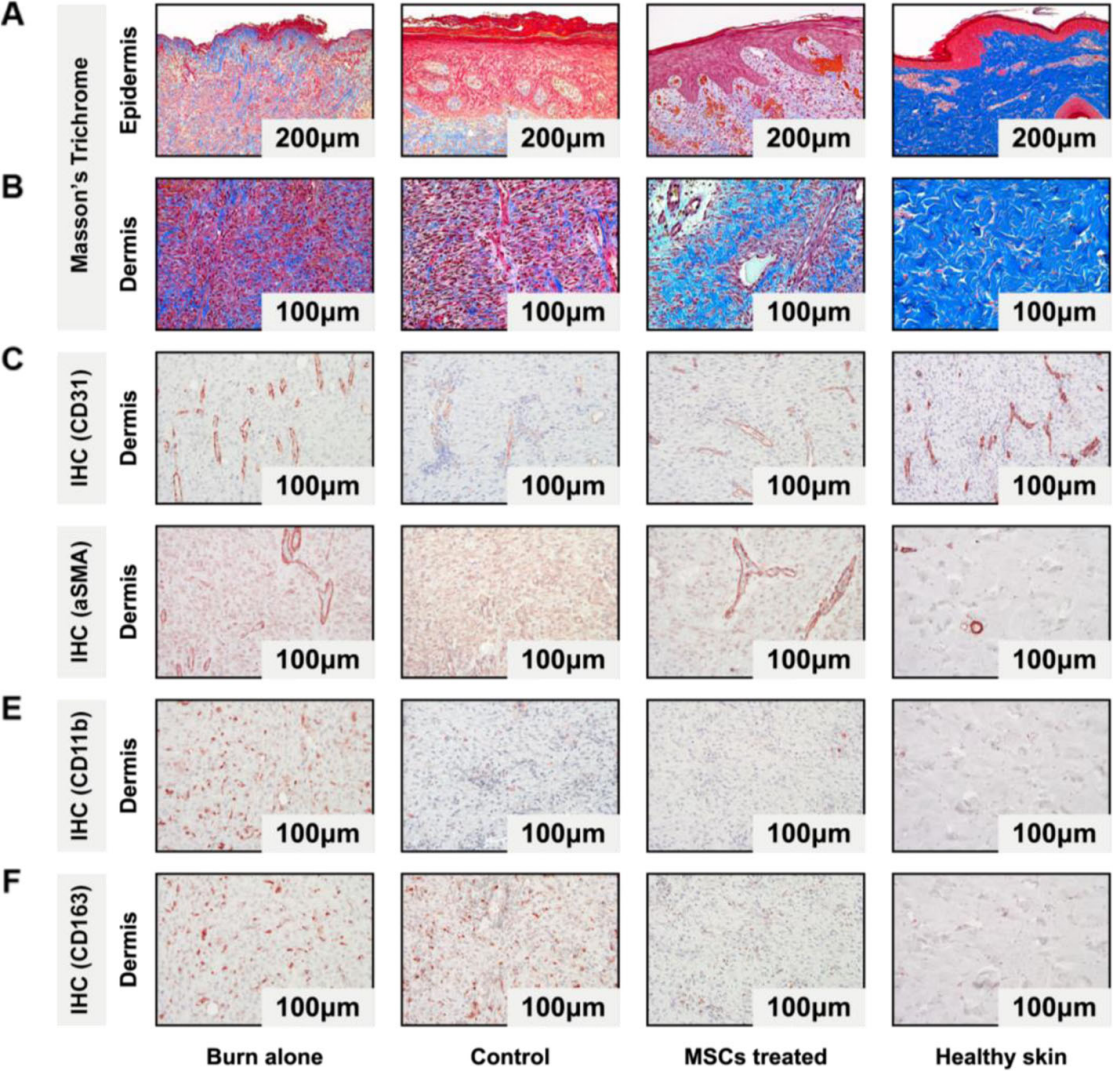
Epidermal and dermal regeneration. **a**, **b** Masson’s trichrome stained epidermis with magnification × 10 and dermis with magnification × 20. **c**–**f** Immunohistochemistry stained dermis with CD31 (**c**), a-SMA (**d**), CD11b (**e**), and CD163 (**f**) with magnification × 20

Histological assessment was also performed 4 weeks after surgery, where tissue biopsies from the wound centers were taken and stained after Masson’s trichrome protocol. For references, healthy porcine skin representing the physiological condition had a median of 165 μm (IQR 159–182 μm), and burn wounds, without any treatment, had a median of 63 μm (IQR 49–75 μm). Hypo- and hyperplasia were defined as inferior or superior epidermal thickness from the interquartile range of the healthy skin. The best regenerated epidermal thickness was achieved from the dose of 200,000 cells/cm^2^ with a median of 157 μm (IQR 99–198), followed by the dose of 40,000 cells/cm^2^ with a median of 189 μm (IQR 132–262), and the dose of 400,000 cells/cm^2^ with a median of 131 μm (IQR 116–149). The acellular control showed a median of 177 μm (IQR 64–383 μm), although it lagged in epidermal regeneration and demonstrated a high range of hypo- and hyperplastic epidermal thickness, where the Integra® scaffold was incompletely degraded by day 28. The DRT was visible in none of the MSC-treated groups. The dose of 5000 cells/cm^2^ showed a median of 284 μm (IQR 205–286 μm) and, according to the reference, was defined as hyperplasia, although the histology showed a very homogenous epidermal regenerated architecture with rete ridges comparable to the other MSC-treated groups (Figs. 1c and 3a, Supplementary Figure 1F).

### Dermal regeneration

Dermal regeneration was evaluated by measuring the collagen density with imaging software, where stained collagen fibers were extracted [45]. All MSCs-treated groups regenerated more collagen compared to the acellular control. Within the dose groups, the dose of 200 cells/cm^2^ reached the highest collagen density (median 639 K, IQR 579–720 K), followed by 200,000 cells/cm^2^ (median 567 K, IQR 527–590 K) and 5000 cells/cm^2^ (median 554 K, IQR 525–605 K). The acellular control reached the lowest collagen density (median 490 K, IQR 464–605 K) (Figs. 1c and 3b).

The tissue was also stained via immunohistochemistry for the endothelial marker CD31, indicative of neovascularization. Measurement was done by counting each vessel with a lumen. Anatomical structure was counted once [46]. All MSC-treated group showed higher neovascularization compared to the acellular control. Within the different dose groups, the dose of 40,000 cells/cm^2^ showed the same vessel count of 17 (IQR 12–17) compared to the healthy skin (median 17, IQR 13–18). Closest to the reference, the dose of 200,000 cells/cm^2^ showed 15 vessels (IQR 12–16), followed by 400,000 cells/cm^2^ with 19 vessels (IQR 15–23). The acellular control regenerated 7 vessels (IQR 6–10) (Figs. 1c and 3c).

In a fibrosis assessment, positive alpha-smooth muscle cells (a-SMA) were stained and measured with imaging software after extraction and an adjusted density assessment [45, 47, 48]. All MSC-treated groups showed a lower positive a-SMA cell density compared to the acellular control (median 412 K, IQR 386–485 K). We found the lowest fibrotic appearance with lowest positive a-SMA count in the 40,000 cells/cm^2^ (median 30 K, IQR 27–39 K) dose group, followed by 200,000 cells/cm^2^ (median 31 K, IQR 29–34 K), and 5000 cells/cm^2^ (median 37 K, IQR 36–40 K) (Figs. 1c and 3d).

The tissue was also stained for positive inflammatory markers. Due to the high cross-reactivity of the antibodies in the pig tissue, it was challenging to find reliable markers for macrophages. A clear signal was found for CD11b and CD163. CD11b is a pan-macrophage marker, which is expressed on a variety of leukocytes and is upregulated on activated cells, including type 1 macrophages [49, 50]. Due to the observation that the tissue showed different states of present inflammatory cells in the remodeling phase on day 28 depending on wound location, the epidermal border region and the dermis was assessed separately to quantify differences. The lowest CD11b-positive cell counts were found in the epidermal border region in the wounds with 40,000 cells/cm^2^ with a median of 8 (IQR 6–11), followed by 200 cells/cm^2^ with a median of 12 (IQR 5–15) and 5000 cells/cm^2^ with a median of 12 (IQR 7–18). The wounds with 400,000 cells/cm^2^ showed a median of 22 (IQR 21–35) and the wounds with 2,000,000 cells/cm^2^ showed a median of 37 (IQR 31–44), which was more compared to the acellular control with a median of 17 (IQR 10–30). Evaluating the dermal region, the dose of 40,000 cells/cm^2^ showed the lowest positive cell count of 7 (IQR 6–11), followed by the dose of 5000 cells/cm^2^ with a median of 9 (IQR 8–10), and 200 cells/cm^2^ with also a median of 9 (IQR 6–16). The acellular control showed a lower median of positive counted cells of 32 (IQR 15–49) compared to the highest dose group with 2,000,000 cells/cm^2^ with a median of 36 (IQR 8–50) (Figs. 1c and 3e).

Along with the pro-inflammatory marker CD11b, the tissue was stained for CD163, which is a marker expressed on anti-inflammatory and pro-repair cells such as type 2 macrophages [51, 52]. In the epidermal border region, all MSC-treated groups showed a lower positive cell count of CD163 positive cells, compared to the acellular control. The wounds with 5000 cells/cm^2^ showed the lowest median of 10 (IQR 9–20), followed by 40,000 cells/cm^2^ with a median of 12 (IQR 8–24), and 200 cells/cm^2^ with a median of 25 (IQR 16–54), than the acellular control with a median of 68 (IQR 48–72). In the dermal part, all MSC-treated groups showed fewer positive cells than the acellular control with a median of 68 (IQR 34–72). Within the different dose groups, we found the lowest positive cell count when treating wounds with 5000 cells/cm^2^ with a median of 8 (IQR 6–13), followed by 40,000 cells/cm^2^ with a median of 12 (IQR 4–29), and 200,000 cells/cm^2^ with a median of 31 (IQR 25–34) (Fig. 1c, 3f).

## Discussion

In our low-to-high MSCs-dose treatment model, where we evaluated 8 wound healing parameters, we show that the low dose of 40,000 cells/cm^2^ regenerates the full-thickness burn excised wounds most efficaciously, followed by an even lower dose of 5000 cells/cm^2^. Third was equally effective at 200 and 200,000 cells/cm^2^ compared to higher dosages up to 2,000,000 cells/cm^2^.

This is an important finding given that previous studies have hypothesized that more cells lead to a better outcome in skin healing [9, 12–16, 32]. MSC cell therapy is a potentially powerful treatment and (autologous) sources are readily and cost-effective available. Therefore, determining cell dosage for clinical trials is essential to preventing therapy failure. Our study with a wide dose range fills a gap with respect to dosage and discusses the effects of future cell-based therapy.

We confirm with our pre-clinical results’ previous findings stating that mesenchymal stromal/stem cell therapy improved macroscopical wound healing with faster epithelialization, reduced scarring, and reduced inflammation. Furthermore, we proved that this beneficial cell therapy with pro-angiogenic and fibroproliferative effects increased collagen formation, increased neovascularization, and reduced fibrosis [11, 30, 53–55]. Additionally, we demonstrate that the newly cellularized MSCs treatment is safe and accelerates wound healing more effectively compared to the acellular control used in clinic.

We hypothesize that the better outcome in the low dose range is explainable due to the very simple adage “the dose makes the poison” and with three underlying mechanisms (based on a publication in *Cell*, of a mathematical model of cell circuits of cell proliferation and death [56]). First, an excessive amount of grafted stem cells, such as 2,000,000 cells/cm^2^, may be proliferating to a maximum consuming space and use all available growth resources. This generates a lack of nutrients and possible hypoxia in the wound environment which would lead to cell death. Massive signaling occurs which needs to be regulated and may take longer until tissue regeneration occurs compared to other cell dosages. It has been shown that MSCs reduce hypoxia-induced apoptosis [57] and additionally showed a beneficial initial inflammatory upregulation in MSCs that prevents hypertrophic scar formation [54, 58–60], which would be in line with our findings. For very low initial cell concentrations, the cell numbers may be declining since the critical threshold of hemostasis is not reached, but the paracrine signals may provide the neighboring cells in the wound bed a very early proliferative healing boost as shown in the results. Given the initial appropriate range of dose, hemostasis can be achieved faster, leading to the most optimal accelerated healing.

However, the explanation why different cell-dosages have varying efficacies might be more complex. The extracellular microenvironment (and the biomaterial as cell carrier itself) is taken into account. For instance, recent studies have shown that the collagen scaffold as MSC carrier leads to inferior wound healing compared to xenografts [61], but the DRT also demonstrated superior healing compared to a soft, fast biodegradable biomaterial [10]. This highlights that the extracellular components also play a detrimental key role in guiding the cells.

This analysis presented here evolved after an unexpected observed low-dose healing phenomenon in an ongoing trial, where we retrospectively analyzed our collected dataset. We therefore recommend for future research to create a dose-model that is translatable and to implement objective scientific methods to determine healing or any outcome measures of interest (Supplementary Material, Table 1).

### Limitations

We did not determine the cell viability, the state of differentiation, or the potential harm of the delivered cells on the in vivo wounds after grafting (of the > 153.9 billion cells). This would have been interesting but not feasible in such large inflicted injuries primarily investigating wound healing (and therefore avoiding wound biopsies for proofing). Each cell manipulation can potentially affect the transplanted cells by inducing down-stream changes [62]. We performed cell sorting for the MSCs surface markers 1 day before surgery and found similar quantities as other researchers found after large scale expansion using UC-MSCs [63]. Before incorporating into the DRT, the cells had a homogenous morphology by adhering on the plastic culture flask before preparation and same-day-surgery.

In comparable cell tracing experiments in Integra®, it was shown that the cells were also no longer detectable [33, 34] after 1 week, using the same cell surface dye [40]. These wounds were excluded in our calculation due to the multiple biopsies needed for analysis. Exact cell tracing using eventually methods such as GFP^+^-tracing to determine cell fate would have been interesting, but this was not our primary focus.

Furthermore, it would have been interesting to take biopsies and investigate the molecular cytokine profile [64] and perform quantitative analysis of paracrine effects [65]. Moreover, we could have investigated the survival in each dose wound and measured hypoxia. However, due to the constraints of the study and the costly time-consuming nature of porcine research, we did not perform these analyses herein. Fine-tuning and optimizing the cell dosage as well as measuring alterations in the cytokine/chemokine profile from various cell concentrations and cell sources might be done in the future in a prospective setting.

For statistical analysis, we tried to cluster MSC doses in our non-parametric dataset to a low-middle-high dose, using generalized estimating equations (GEEs) models. This estimation model accounted for dependencies among the data introduced by multiple wounds on the same pig (“healing capacity of each individual”), including the treatments (7 treatments, 2 references), which were performed on 3–7 different pigs, between 3 and 12 times. However, due to the low N the determination between the clusters would have neglected the third best results of 200,000 cells/cm^2^. Additionally, the (extreme) lowest dose of 200 cells/cm^2^ and (extreme) highest dose of 2,000,000 cells/cm^2^ would have not been included in the model, due to the sample size. Therefore, we decided to show the descriptive data rather than the misleading GEE significance.

### Future directions

Our results in this pre-clinical study highlight two directions for future research. First, the dose-model can be translated to humans for potential autologous MSCs treatment trials, due to the principal similar skin structure from pigs to humans. MSCs treatment works at any dosing—from low to high; however, it is crucial to determine the most optimal cell therapy for patients, for partial and full-thickness regeneration, for acute and chronic wound healing.

Second, an off-the-shelf therapy using the released paracrine products will be successful, if the optimized “cell dose cocktail” is quantitatively determined. This therapy can then act as a frontier in regenerative medicine.

## Conclusion

This study gives new insights based on a cell-dose-dependent wound healing model in full-thickness skin regeneration and shows most efficacy in low doses compared to higher dosages. This is a decisive finding for future investigations using stromal/stem cells. To the best of our knowledge, there are no comparisons available that including such a wide range of doses in such a large pig animal trial. This cell-dose model can be translated and implemented to innovative, regenerative stem cell therapy.

## Supplementary Information

**Additional file 1: Figure S1.** Schematic purpose of the DRT Integra®, cell incorporation into the DRT, cell viability after labeling cells with DiO, DRT cell infiltration, DiO detection via flow cytometry, DRT Remodeling. (A) Schematic purpose of the DRT. Excision and removal of the wound tissue, grafting of the DRT for cell ingrowth and remodeling until the protecting silicon layer is removed. (B) Cellularization of the DRT. (1) Stained after 12 h with ActinGreen and DAPI, horizontal view. (2) Vertical view, green channel, stained with ActinGreen. (3) Seeding depth 123 ± 21 μm (SEM, *N* = 3), in the 1.3 mm thick scaffold. (C) Live-Dead-Staining 12 h after flow cytometry and DiO-labeling, confocal microscope, magnification × 20, as followed: (1, 4) Live cells (calcein) (green channel), (2, 5) Dead cells (EthD) (red channel), (3, 6) Merged. (D) H&E stained DRT after tissue biopsy and tissue preparation, on day 4 and 7, magnification × 20. Dark yellow colored line indicates the upper boarder from the DRT. The DRT is violet stained (as seen in both images on day 4). The brown line at the left bottom image border indicates the DRT scaffold structure. (E) Flow cytometry on day 7 after tissue preparation with a double positive cell signal of a cell surface dye (DIO) on CD90+ cells. (F) Masson’s Trichrome Staining of Histology on day 7, 14, 21 and 28. Magnification × 10. **Figure S2.** Line of best fit for cell dose concentration per parameter. Each graph illustrates a regression (of order 2) with the line-of-best-fit; x-axis is cell dose concentration shown on a logarithmic scale and on the y-axis are the parameters. The 95-confidence-interval is the area in light blue. (Not normalized, raw data set.). **Table S1.** Future directions, outlook and other research questions. Potential associated explanations for different outcome in the presented data set.

## Data Availability

The datasets generated and analyzed during the current study are available from the corresponding author on reasonable request.

## Abbreviations

MSC: Multipotent mesenchymal stromal/stem cell
UC-MSCs: Umbilical cord mesenchymal stromal/stem cells (UC-MSCs)
DRT: Dermal regeneration template
