# Correction to: Skin regeneration is accelerated by a lower dose of multipotent mesenchymal stromal/ stem cells—a paradigm change

**DOI:** 10.1186/s13287-021-02314-9

**Published:** 2021-04-30

**Authors:** Gertraud Eylert, Reinhard Dolp, Alexandra Parousis, Richard Cheng, Christopher Auger, Magdalena Holter, Ingrid Lang-Olip, Viola Reiner, Lars-Peter Kamolz, Marc G. Jeschke

**Affiliations:** 1 Sunnybrook Research Institute, Sunnybrook Health Sciences Centre, Toronto, Canada; 2 Division of Plastic, Aesthetic, Reconstructive Surgery, Medical University of Graz, Graz, Austria; 3 Institute of Medical Science, University of Toronto, Toronto, ON Canada; 4 Department of Psychiatry, Queen’s University, Kingston, Canada; 5 Institute of Biomaterials and Biomedical Engineering, University of Toronto, Toronto, Canada; 6 Institute of Biostatistics, Medical University of Graz, Graz, Austria; 7 Division of Cell Biology, Histology, Embryology, Gottfried Schatz Research Center, Medical University of Graz, Graz, Austria; 8 Coremed- Centre for Regenerative Medicine, Joanneum Research Forschungsgesellschaft mbH, Graz, Austria; 9 Ross Tilley Burn Centre, Sunnybrook Research Institute, Sunnybrook Health Sciences Centre, Toronto, Canada; 10 Division of Plastic and Reconstructive Surgery, Department of Surgery, Faculty of Medicine, University of Toronto, Toronto, Canada; 11 Department of Surgery, Division of Plastic Surgery, Department of Immunology, Director Ross Tilley Burn Centre, Sunnybrook Health Sciences Centre, Sunnybrook Research Institute, 2075 Bayview Ave., Toronto, M4N 3M5 Canada

**Correction to: Stem Cell Res Ther 12, 82 (2021)**

**https://doi.org/10.1186/s13287-020-02131-6**

The original article [1] contained an error mistakenly introduced by the production team whereby affiliations 8 and 11 displayed incorrect city and country information. This has since been corrected.
