# Supplementary material for: Skin regeneration is accelerated by a lower dose of multipotent mesenchymal stromal/stem cells—a paradigm change
Source: Stem Cell Res Ther. 2021 Jan 25;12:82. doi: 10.1186/s13287-020-02131-6 (PMC7831169; doi:10.1186/s13287-020-02131-6)
Supplement: Supplementary file 1 — Additional file 1: Figure S1. Schematic purpose of the DRT Integra®, cell incorporation into the DRT, cell viability after labeling cells with DiO, DRT cell infiltration, DiO detection via flow cytometry, DRT Remodeling. (A) Schematic purpose of the DRT. Excision and removal of the wound tissue, grafting of the DRT for cell ingrowth and remodeling until the protecting silicon layer is removed. (B) Cellularization of the DRT. (1) Stained after 12 h with ActinGreen and DAPI, horizontal view. (2) Vertical view, green channel, stained with ActinGreen. (3) Seeding depth 123 ± 21 μm (SEM, N = 3), in the 1.3 mm thick scaffold. (C) Live-Dead-Staining 12 h after flow cytometry and DiO-labeling, confocal microscope, magnification × 20, as followed: (1, 4) Live cells (calcein) (green channel), (2, 5) Dead cells (EthD) (red channel), (3, 6) Merged. (D) H&E stained DRT after tissue biopsy and tissue preparation, on day 4 and 7, magnification × 20. Dark yellow colored line indicates the upper boarder from the DRT. The DRT is violet stained (as seen in both images on day 4). The brown line at the left bottom image border indicates the DRT scaffold structure. (E) Flow cytometry on day 7 after tissue preparation with a double positive cell signal of a cell surface dye (DIO) on CD90+ cells. (F) Masson’s Trichrome Staining of Histology on day 7, 14, 21 and 28. Magnification × 10. Figure S2. Line of best fit for cell dose concentration per parameter. Each graph illustrates a regression (of order 2) with the line-of-best-fit; x-axis is cell dose concentration shown on a logarithmic scale and on the y-axis are the parameters. The 95-confidence-interval is the area in light blue. (Not normalized, raw data set.). Table S1. Future directions, outlook and other research questions. Potential associated explanations for different outcome in the presented data set. [file 13287_2020_2131_MOESM1_ESM.docx]

**SUPPLEMENTARY MATERIAL**


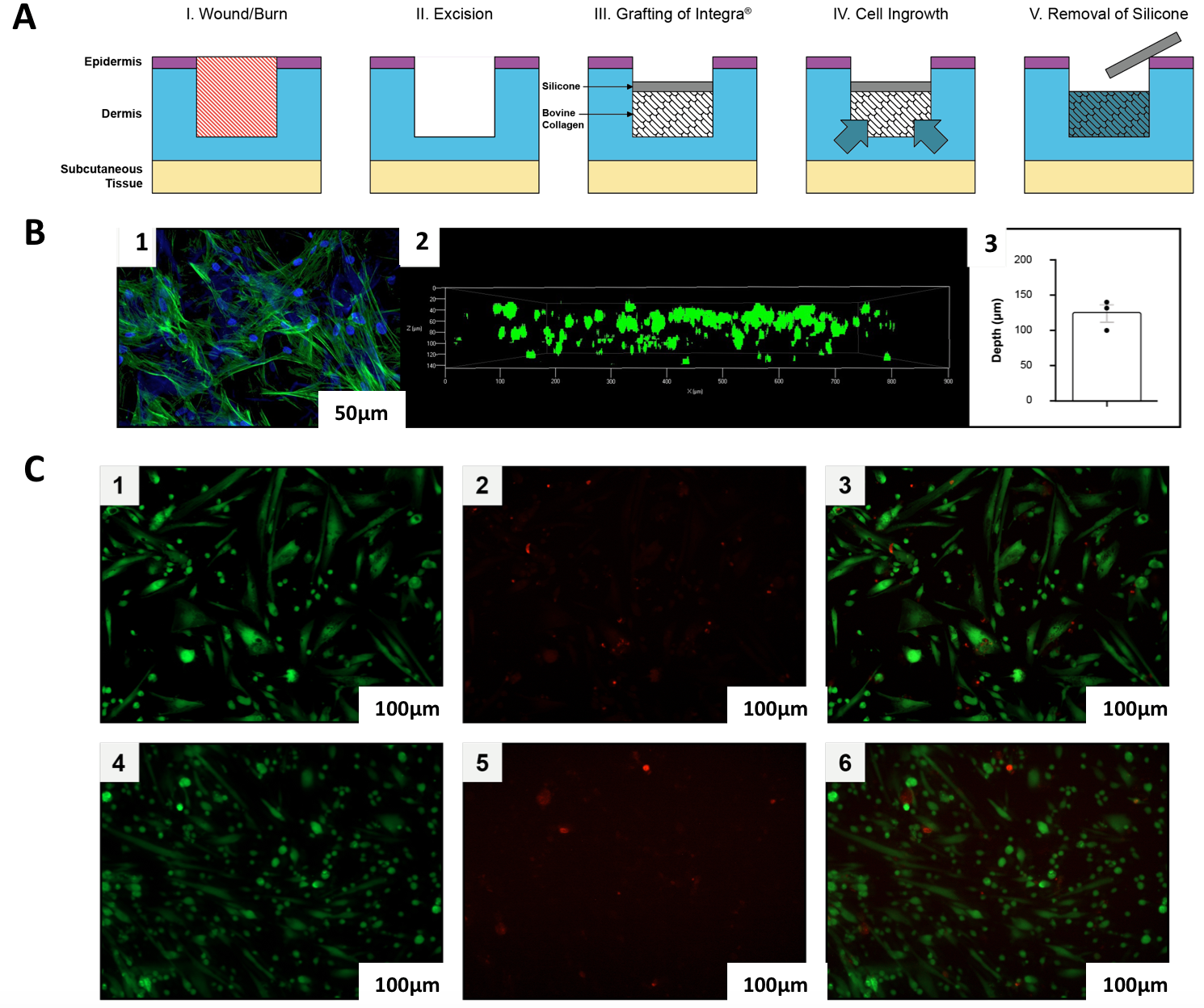

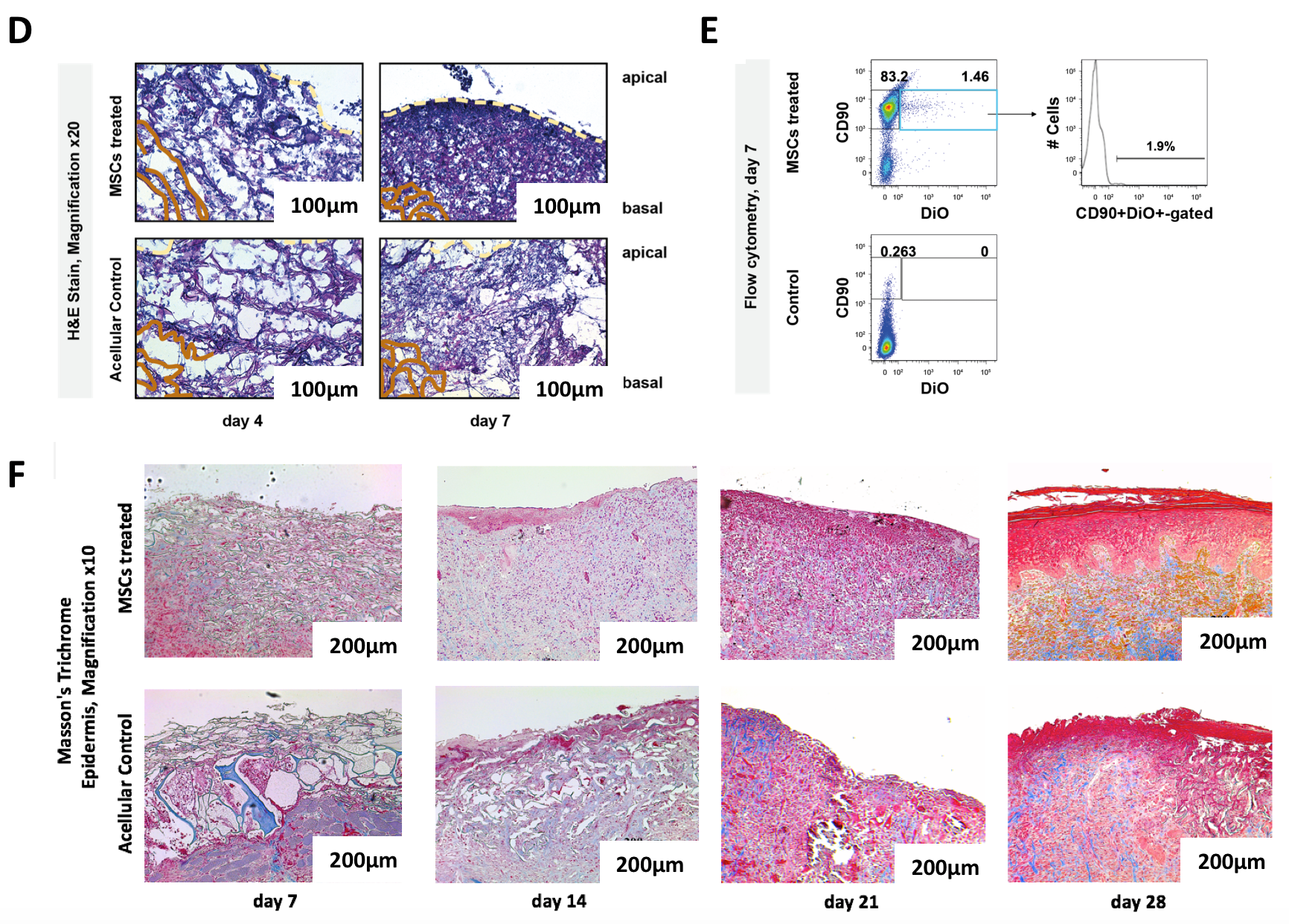


**Supplementary Material Figure 1. Schematic purpose of the DRT Integra®, cell incorporation into the DRT, cell viability after labeling cells with DiO, DRT cell infiltration, DiO detection via flow cytometry, DRT Remodeling.** (A) Schematic purpose of the DRT. Excision and removal of the wound tissue, grafting of the DRT for cell ingrowth and remodeling until the protecting silicon layer is removed. (B) Cellularization of the DRT. (1) Stained after 12h with ActinGreen and DAPI, horizontal view. (2) Vertical view, green channel, stained with ActinGreen. (3) Seeding depth 123 ± 21 µm (SEM, N=3), in the 1.3mm thick scaffold. (C) Live-Dead-Staining 12 hours after flow cytometry and DiO-labeling, confocal microscope, magnification x20, as followed: (1, 4) Live cells (calcein) (green channel), (2, 5) Dead cells (EthD) (red channel), (3, 6) Merged. (D) H&E stained DRT after tissue biopsy and tissue preparation, on day 4 and 7, magnification x20. Dark yellow colored line indicates the upper boarder from the DRT. The DRT is violet stained (as seen in both images on day 4). The brown line at the left bottom image border indicates the DRT scaffold structure. (E) Flow cytometry on day 7 after tissue preparation with a double positive cell signal of a cell surface dye (DIO) on CD90+ cells. (F) Masson`s Trichrome Staining of Histology on day 7, 14, 21 and 28. Magnification x10.


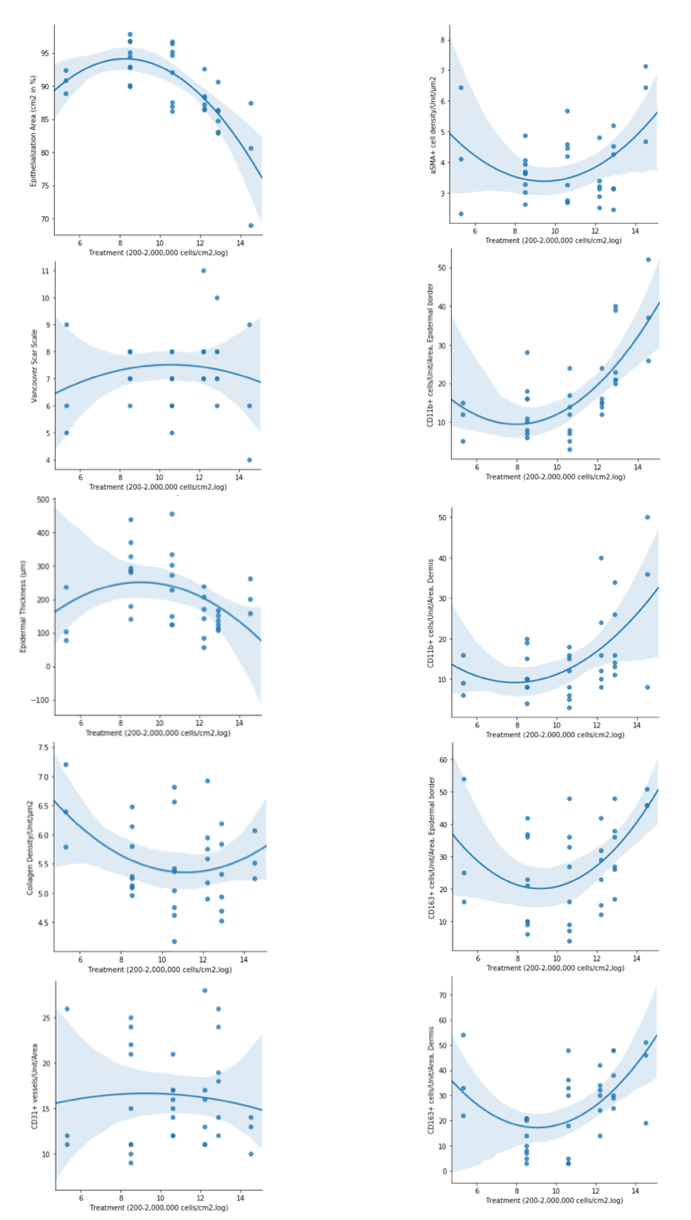


**Supplementary Material Figure 2. Line of best fit for cell dose concentration per parameter.** Each graph illustrates a regression (of order 2) with the line-of-best-fit; x-axis is cell dose concentration shown on a logarithmic scale and on the y-axis are the parameters. The 95-confidence-interval is the area in light blue. (Not normalized, raw data set.)

| **Supplementary Material Table 1.** | | |
| --- | --- | --- |
| **Intracellular** | Cell source | Juvenile UC-MSCs vs. adult skin derived, precursor vs. progenitor cells, quiescence of (adult) cells, level of differentiation of the transplanted cells |
|  | Cell type | Not long Cultured vs. long cultured cells (with already initiated downstream differentiation) |
|  | Cell state | level of internal cell energy and available intracellular nutrients/resources, internal hypermetabolic cell stress levels and mechanism to cope with cell stress, eg. mitochondrial status (ATP, ADP, glycolytic function, mitochondrial function), strategies to cope with hypoxia, mechanism to cope with (nutrient, energy) starvation, intracellular mechanism to cope with (outside/downstream) cell competition, cell cycle phase of (transplanted) cells, regenerative potential of the transplanted cells |
| **Extracellular** | (Host) Environment | Burn (inflicted) wounds, wound bed, host tissue/viability, preconditioned host tissue, (already) “homed” repair cells at the wounded side eg. migrated bone-marrow stem cells, bandage/dressing, warmth/temperature of the wound, manipulation of wounds, healing potential such as immobilization of the wound (sheer/mechanical stress, range-of-motion of wounds) |
|  | Cell Delivery, Carrier | Quality of cell delivery/grafting/transplantation/setting, cell carrier, (traumatic) cell transplantation |
|  | Biomaterial | Environment for cells, eg. soft, stiff material, nutrients available, (space for) cell adherence, cell competition, triggers for cell-cell interaction, potential of biodegradability of the biomaterial, external mechanical stress for cells, migration from cells from the host toward the wound dressing, duration of wound dressing onto wounds (dressing changes, disinfection of wounds, toxicity of dressing changes) |
|  | Cell Integration and Migration | Host Tissue and cells distance (cell-adherence, cell integration, migration), orchestrate cutaneous wound healing cascade including eg. a potential healing environment, nutrients for cells, accurate temperature for cells to migrate/proliferate, outside/wound triggers of the host (chemokines, cytokines), cell environment in the biomaterial, cell-competition, toxicities of/from the host tissue (burn, preconditioned wound, repair potential) |
|  | Cell Therapy (Quantity, Dose) | Quality of cell therapy, contamination, cell-cell-interactions, nutrients, (overload) on cytokines/chemokines from other cells - toxicity, paradoxical cell signaling (from to many, to few cells) |

**Supplementary Material Table 1. Future directions, outlook and other research questions.** Potential associated explanations for different outcome in the presented data set.
